# Apixaban Overdose with Massive Bleeding and Anti-Xa Levels

**DOI:** 10.1007/s13181-026-01137-5

**Published:** 2026-04-13

**Authors:** Rebecca Kusko, Jami Hagemann, Daniel McCabe, Joshua Trebach

**Affiliations:** 1 Department of Emergency Medicine, University of Iowa Carver College of Medicine, Iowa City, IA 52242 USA; 2 Iowa Poison Control Center, Sioux City, IA 51101 USA; 3 College of Pharmacy, University of Iowa, Iowa City, IA 52242 USA

**Keywords:** Apixaban, Direct Oral Anticoagulant, Overdose, Hemorrhage, Case Report

## Abstract

**Introduction:**

While use of factor Xa inhibitors has increased, there are relatively few cases of overdose reported. Clinically relevant bleeding is often absent, and the behavior of drug levels in overdose is not fully agreed on.

**Case Report:**

A 70-year-old male presented after a polysubstance overdose including at least 250 mg of apixaban with massive gastrointestinal (GI) hemorrhage and parafalcine and tentorial subdural hematoma. He was treated with 1 gram tranexamic acid (TXA), anti-inhibitor coagulant complex (FEIBA), and massive transfusion. No further episodes of bleeding were observed. Anti-Xa levels were trended until undetectable.

**Discussion:**

Bleeding from an overdose of factor Xa inhibitors is a relatively rare occurrence; however, this case illustrates that life threatening hemorrhage can occur and may require aggressive supportive care. Anti-Xa levels demonstrated a first order pattern of elimination even at markedly supratherapeutic concentrations, consistent with several prior case reports.

## Introduction

Over the past 15 years, direct oral anticoagulants, particularly factor Xa inhibitors, have largely replaced warfarin as the preferred therapeutic option for common thromboembolic conditions [1]. Their benefit of fixed dosing and lack of need for routine laboratory testing make them more appealing to patients. Although the use of factor Xa inhibitors is increasing, there are few documented cases of overdoses with both corresponding anti-Xa level trends and significant bleeding observed on physical exam. We present, in accordance with the CARE guidelines (https://www.care-statement.org), a case of a patient with significant bleeding and hemodynamic instability in the setting of apixaban overdose with accompanying anti-Xa levels.

## Case

A 70-year-old Caucasian 104.4 kg male with a past medical history of coronary artery disease with recent percutaneous coronary intervention for ST-segment elevation myocardial infarction (STEMI) and atrial fibrillation anticoagulated on apixaban in addition to anti-platelet agents aspirin 81 mg daily and clopidogrel 75 mg daily for 7 days of triple therapy post stent placement, presented from home to a tertiary academic medical center emergency department (ED) via emergency medical services (EMS) following an intentional ingestion of at least 250 mg apixaban, in addition to ingestion of 10–15 tabs of acetaminophen (APAP) of unknown strength, 10–15 tabs of ibuprofen of unknown strength, and an unspecified wart removal solution. On arrival to the ED, EMS reported that the patient had been found by bystanders covered in hematemesis and melena. EMS reported that they had administered a 1-liter intravenous fluid bolus and 1 gram of intravenous tranexamic acid (TXA) en route. On initial exam in the ED, patient was pale and fatigued, covered in hematemesis, shivering, in moderate distress, but following commands and answering questions with a GCS of 15. He disclosed that in addition to the ingestion of the above medications, he also intentionally cut himself. Physical examination revealed a 1 cm superficial laceration without active bleeding. No additional signs of trauma were noted. Vital signs were notable for blood pressure of 67/50 mmHg, heart rate 120 beats/minute, respiratory rate 24 breaths/minute, temperature 35.8 °C, oxygen saturation 93% via pulse oximetry on room air.

Initial laboratory tests were notable for white blood cells of 23,100/mm^3^ (reference range 3,700–10,500/mm ^3^) hemoglobin 7.1 g/dL (from 12.0 g/dL four days prior) (reference range 11.9–15.5 g/dL), lactic acid 7.5 mmol/L (reference range 0.5–2.2 mmol/L), INR 1.9 (reference range 0.8–1.2), PT 19 s (reference range 9–12 s), fibrinogen 284 mg/dL (within normal limits; reference range 194–448 mg/dL), venous pH 7.26 (reference range 7.33–7.43), bicarbonate 16 mEq/L (reference range 22–29 mEq/L), blood urea nitrogen (BUN) 93 mg/dL (reference range 10–20 mg/dL), creatinine (Cr) 2.4 mg/dL (reference range 0.67–1.17 mg/dL), eGFR 28 mL/min/1.73 m2, calcium 7.8 mg/dL (reference range 8.5–10.5 mg/dL), and high-sensitivity troponin 120 ng/L (1,500 eight days prior at time of STEMI, reference range less than or equal to 22 ng/L). Liver enzymes were obtained and were within normal limits; AST 29 U/L (reference range 0–50 U/L), ALT 21 U/L (reference range 0–50 U/L), total bilirubin 0.2 mg/dL (reference range less than or equal to 1.2 mg/dL), alkaline phosphatase 52 U/L (references range 40–129 U/L). Albumin was mildly low at 3.0 g/dL (reference range 3.4–4.8 g/dL). An anti-Xa level was obtained with the initial labs at presentation (~ 10 h post ingestion). The result was above the detectable limit, but after dilution a quantitative value of 1,498 ng/mL was measured (no defined reference range). Our method was developed and validated in-house and the methodological details have not been published. We utilize the Siemens Innovance Anti-Xa reagent calibrated for apixaban using calibrators from Hyphen Biomed. The assay is run on a Siemens CS-5100 analyzer. [2, Personal Communication]. On presentation, serum acetaminophen concentration was below the level of detection (limit of detection is 5 ug/mL, this places patient well below the threshold to treat with N-acetylcysteine based on the time of ingestion) and serum salicylate concentration was 2.7 mg/dL (no reference range defined, but therapeutic range given as under 30 mg/dL). An ethanol level was not obtained.

Computed tomography (CT) imaging of the head was notable for thin acute right parafalcine and tentorial subdural hematoma. No acute trauma was reported, however, the patient was reported to have been found lying down, suggesting a fall is possible. CT imaging of the chest/abdomen/pelvis was without acute process. The patient received an additional 500 mL bolus of normal saline, and a massive transfusion protocol was initiated. The patient was transfused with 3 units of packed red blood cells, 1 unit of fresh frozen plasma, and 1 unit of platelets. Anti-inhibitor coagulant complex (FEIBA) was administered (5416 units, 50U/kg) in the ED 11.5 h post ingestion in accordance with institutional anticoagulant reversal protocol, and 10 mg Vitamin K was administered IV in the ICU 14.7 h post ingestion. Although vitamin K does not directly reverse apixaban, it was administered empirically early in care prior to confirmation of isolated factor Xa inhibitor effect. Activated charcoal was not administered due to the active GI bleed, tenuous airway status given evidence of recent vomiting, and because several hours had elapsed since the time of ingestion.

**Fig. 1:**
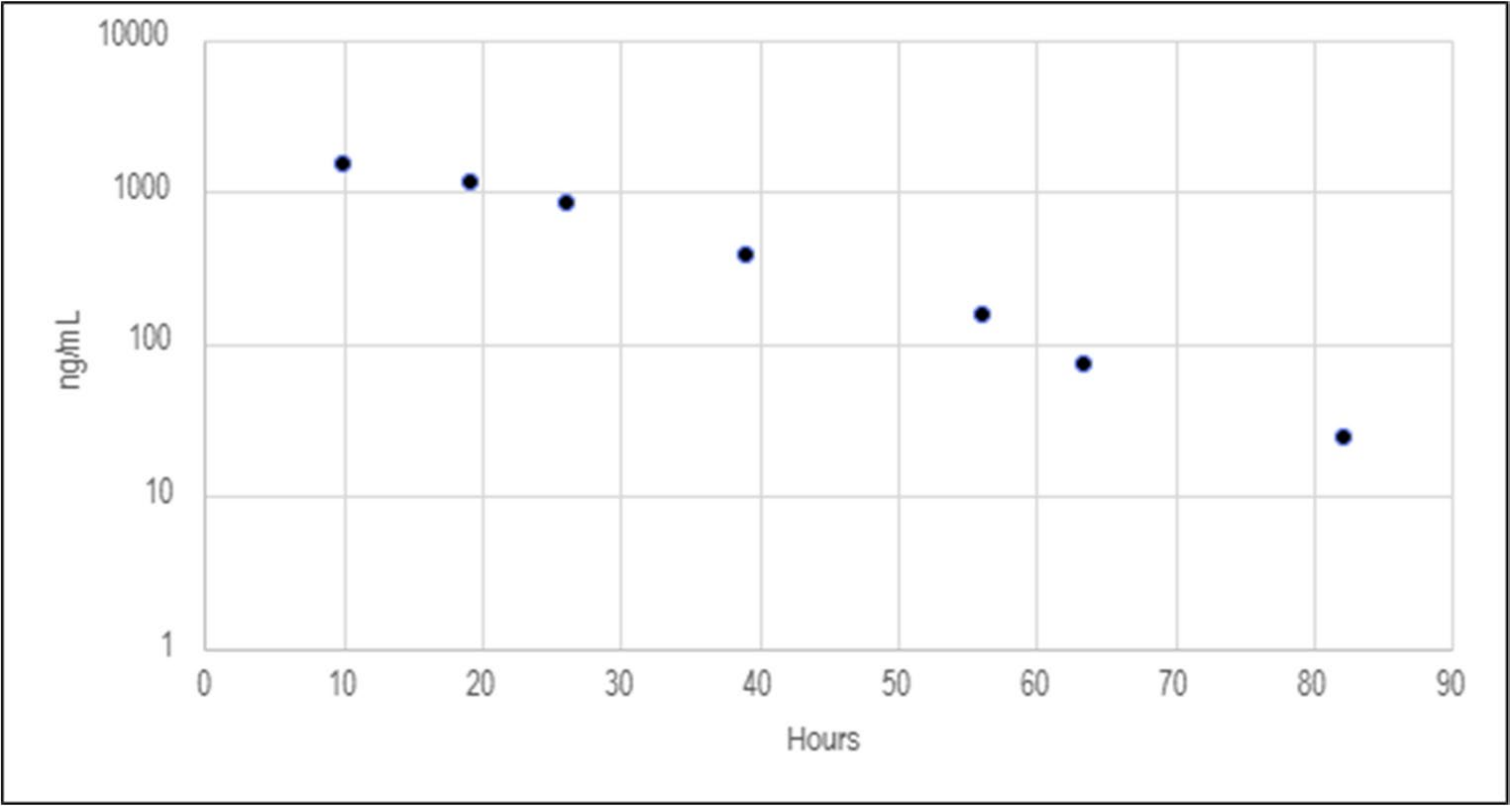
Serial anti-Xa levels post ingestion Created using Microsoft Excel

After completion of the massive transfusion protocol, the patient’s repeat hemoglobin was 12.5 g/dL. Hemoglobin levels were monitored and had decreased to 9.5 g/dL by hospital day (HD) 13 at the time of discharge, without evidence of rebleeding after initial arrival. Anti-Xa levels were trended throughout the patient’s hospitalization (Fig. 1; Table 1).

**Table 1:** Timeline of events

Time (hrs)	Anti-Xa Level (ng/mL)	Event
0		Overdose
8		Call to EMS
		EMS administered 1 L IVF, 1 g TXA
9.8		ED arrival
10.0	1498	Coagulation panel drawn (PT 18 s, INR 1.9, PTT 30 s)
10.1		MTP order placed, 3 units RBC, 1 unit FFP, 1 unit Plt
11.5		5416 units FEIBA
14.7		10 mg Vitamin K
19.1	1138.5	
26.0	834	
35.3		Clopidogrel restarted
39.0	389	
56.1	157	
63.4		Aspirin restarted
63.5	76	
82.1	25	

Abbreviations used in the table are: *EMS* emergency medical services, *IVF* intravenous fluids, *TXA *transexamic acid, *ED* emergency department, *MTP *massive transfusion protocol, *RBC* red blood cells, *FFP* fresh frozen plasma, *Plt *platelets, *FEIBA* anti-inhibitor coagulant complex

Creatinine levels had risen to 2.40 mg/dL from a baseline of 1.1 mg/dL prior to ED presentation. These fell to 1.44 mg/dL by HD2 and returned to baseline by HD3.

Clopidogrel 75 mg was restarted on HD2, and aspirin 81 mg was restarted on HD 3 due to risk for development of stent-related thrombosis. Repeat imaging of the subdural hematoma was recommended in 6 h by neurosurgery, as well as levetiracetam load of 1000 mg followed by levetiracetam 500 mg twice daily for 7 days. The follow up CT brain demonstrated stability, and no further decompensation occurred during the remainder of hospitalization. Apixaban 5 mg twice daily was restarted based on cardiology recommendations to continue clopidogrel, and resume apixaban while stopping aspirin once cleared to do so by neurosurgery. The primary team ultimately continued clopidogrel, stopped aspirin, and resumed apixaban on HD7 based on the lack of rebleeding episodes following a shared decision-making conversation that day and the day prior. No additional anti coagulants were given prior to this for thromboembolism prophylaxis. No thrombotic event was documented during hospitalization or within 6 months of follow-up.

The gastrointestinal (GI) bleed was treated with 40 mg IV pantoprazole in the ED, and the patient was continued on 40 mg IV pantoprazole twice daily until the afternoon of HD3, when he was transitioned to oral pantoprazole. Endoscopy was considered; however, patient was initially deemed too unstable, and the risk of endoscopy too high in the setting of supratherapeutic anticoagulation. Because there was no recurrence of bleeding following stabilization, the source of the bleed was suspected to be mucosal on repeat GI evaluation, and endoscopy was ultimately not performed during this hospitalization. Patient was continued on oral pantoprazole, 40 mg twice daily, for 8 weeks following discharge. Consent for publication of this case was obtained and provided to the journal in accordance with JMT policy.

## Discussion

We describe a case of life-threatening hemorrhage in the setting of apixaban overdose, with serial anti-Xa levels obtained throughout hospitalization. Two prior case reports describe apixaban overdoses complicated by bleeding, one with a fall resulting in subarachnoid hemorrhage (SAH) [3] and one fatal case due to gastrointestinal/internal hemorrhage [4]. Other reports have documented apixaban overdoses without clinically significant bleeding [1, 5–12] (Table 2).

**Table 2:** Prior case reports and description of kinetics, treatments, and bleeding [1, 3–12]

First Author	Year	Reported Dose (mg)	Coingestions	Bleeding	Reversal Agent	Description of Elimination Kinetics	Half Life (if reported)
Barton	2016	200	Ramipril, bisoprolol, atorvastatin, colchicine, magnesium, acetaminophen, codeine, phenylephrine, alcohol	Minor (from central line site)	Vitamin K, Prothrominex-vf (Factors II, IX, X), given after initial lab	Linear	7.4
Leiken	2017	300	None	None	None	Non linear	
Mast	2017	280	None	Minor (small wound left elbow)	None	Linear	8
Guadarrama	2018	200	None	None	None	Biphasic	
Ilicki	2018	210	Levothyroxine, amitriptyline	None	None	Not described	
Franck	2019	300	Acetaminophen, oxazepam	None	None	Linear	10.8
Launay	2020	40 (23 mo old)	Digoxin	None	None	Linear	8.2
Delrue	2022	300	Acetaminophen, zopiclone, bisoprolol	None	None	Non Linear First Order	36.5
		230	Amlodipine, bisoprolol, hydrochlorothiazide, acetaminophen, atorvastatin, clomipramine, potassium	None	None	Non Linear First Order	22
		Unknown	Bisoprolol, ramipril, atorvastatin, eplerenone	None	None		
Jenniches	2023	275	Carvedilol, atorvastatin, amlodipine, ethanol	Traumatic ICH	FEIBA (multiple doses), FFP, TXA, Andexanet al.fa	Not trended	
Kintz	2023	200 (autopsy)	Diazepam, flecainide, mianserine, alcohol	GI (massive), liver, kidneys	None	Not trended	
Harmouche	2024	300–350	None	None	AC, FFP	Linear	14

In spite of the bleeding, anti-platelet therapy was continued early in the hospital course, clopidogrel was restarted on HD2 and aspirin on HD3, due to the recent placement of a cardiac stent, and high risk of stent thrombosis. As the patient remained hemodynamically stable following resuscitation, with no further bleeding events reported, the risk of stent thrombosis was deemed to be higher than the risk of rebleeding. In a similar fashion, after several days of hospitalization during which no further bleeding events were observed, aspirin was stopped and apixaban restarted based on cardiology consultant’s preference for apixaban and clopidogrel as their ultimate agents of choice. The decision to restart apixaban was ultimately made by the primary team after 2 days of shared decision-making discussion with the patient; by this point anti-Xa levels were undetectable, suggesting the patient was not anticoagulated at all when apixaban was restarted.

Had the patient rebled, treatment with additional doses of FEIBA would have likely been pursued given the initial good response, with treatment following clinical effect rather than specific factor Xa levels. Our experience with hemostasis, however, contrasts with the case described by Jenniches et al. [3], where they report continued bleeding of a traumatic SAH despite multiple doses of FEIBA; andexanet alfa was ultimately given in that case along with operative management. The difference may be due in part to the different locations of bleeding – a large subarachnoid clearly due to trauma in their case versus a small subdural hemorrhage with uncertain / unlikely trauma and a large GI bleed ultimately thought to be mucosal in our case. The difference could indicate varied sizes of vessel compromised, with the traumatic bleed causing a larger vessel to be compromised that was less able to clot even after reversal. Another theory reflects that the patient in the Jenniches case was reported to have significant daily alcohol use, and had a dramatically elevated INR, which was not present in our patient even before reversal; it is possible the patient in the Jenniches case potentially had a baseline coagulation abnormality due to impaired factor synthesis in addition to the apixaban she took.

FEIBA was chosen as the reversal agent in our case due to institutional formulary, which is based on the theoretical benefit of containing activated factor VII, which allows the clotting cascade to bypass the inhibited factor Xa [13]. However, clinical series have shown similar effectiveness between PCC and FEIBA, though FEIBA may allow a smaller dose to be used [13], and both it and PCC are recommended by expert guidelines [14].

In overdose, drug kinetics may deviate from the typical first-order pattern observed with therapeutic dosing. However, several reports suggest that, beginning a few hours after ingestion, apixaban elimination continues to approximate first-order kinetics even at markedly supratherapeutic concentrations. This case contributes to the growing literature on apixaban overdose, particularly regarding the trajectory of anti-Xa levels, which have been shown to correlate linearly with apixaban concentrations [5, 6, 15]. Reported cases showing linear kinetics of apixaban concentration include: a patient with a markedly elevated apixaban level who received fresh frozen plasma (FFP) [7]; a 53-year-old who received vitamin K and three-factor prothrombin complex concentrate [8]; a 67-year-old who demonstrated delayed peak levels but linear elimination [6]; and both a 48-year-old [5] and a 23-month-old [9] who received no reversal agents. The case series by Delrue et al. of three patients with apixaban overdoses plotted apixaban levels in two patients. While they report half-lives (and Ke), implying first order kinetics, these are greatly prolonged when compared to our values and those of other case reports. Within the discussion, the authors comment on this, stating that while most of their case series had half-lives that were in line with therapeutic values (given in this paper as 8 to 15 h for apixaban), these two patients did not, and note that they suffered from “marked cardiovascular failure” which is attributed to the co-ingested cardiac drugs in these cases. One of these two patients is also described as having also suffered renal failure, which could further contribute to delayed clearance [1]. Guadarrama et al. [10] describe the decline in levels in their case as ‘biphasic’, however, they appear to mean by this that the absolute value declines more quickly when concentrations are higher, and later state that the decay pattern best fits exponential decay, and describe it with a first order equation. Leiken [11] does describe a variable half-life, however, this may be due to limited data points, as only 3 were collected. Ilicki et al. [12] does not describe kinetics in the new case they present, however, they do comment on amitriptyline co-ingestion that may have delayed absorption, and may account for the first time point actually showing a lower apixaban level then the second time point.

Our patient presented with an anti-Xa level of 1498 ng/mL. For reference, the range for a 5 mg twice-daily dose for stroke prevention is 91–321 ng/mL for peak concentration, and 41–230 ng/mL for trough concentration [16]. Our patient’s anti-Xa levels declined steadily throughout hospitalization and were well-modeled by first-order kinetics, with an estimated half-life of approximately 11.95 h (Ke is 0.058). The observed half-life and Ke are similar to the half-life and Ke of 12 h and 0.058 reported in the package insert [17] and contribute to the varying reported half-lives seen in the literature in overdose. Possible reasons for the similarity to therapeutic values could be that apixaban elimination pathways could have remained unsaturated in this case despite the overdose, peak apixaban concentration was not captured and we had fallen back into linear elimination by the time measurement was started, or that the highly protein bound nature of apixaban worked as a buffer for the free drug concentration. Coingestants of acetaminophen, ibuprofen, and wart removal solution (likely salicylic acid based on common products and detectable salicylate level) would not be expected to affect clearance, as none of these agents act on CYP 3A4 or p-glycoprotein, which are involved in apixaban metabolism and elimination [17]. Changing patient levels of factor Xa would also not be expected to influence measurement; after consulting our lab director, they assured us that anti-Xa assays add an excess of factor Xa in the reaction and should not be influenced by fluctuations in factor Xa in the patient sample [Personal Communication].

## Limitations

Our report has several limitations. First, we describe only a single case of apixaban overdose with massive bleeding, limiting generalizability. While the bleeding in this case may be due to apixaban overdose, we cannot rule out additional trauma that may have contributed to the bleeding, or preexisting gastrointestinal pathology that would have caused massive bleeding without the apixaban overdose present. An additional limitation is that we measured apixaban concentration using anti-Xa levels. While these have been shown to correlate linearly with drug concentration [5, 6, 15], our hospital lab relates the measured anti-Xa level to drug concentration based on in-house calibration, rather than a verified standard. Further limitations exist with the history provided in this case, which was provided entirely by the patient and may not be reliable regarding dose of medications ingested, timing of medications ingested, or if other medications were ingested. If the history were not accurate, it could significantly impact interpretation of drug and/or assay levels.

## Conclusion

This case describes a large apixaban overdose resulting in life threatening gastrointestinal hemorrhage requiring transfusion of multiple blood products and systemic hemostatic agents. Serial anti-Xa levels demonstrated continued first order kinetics even at markedly elevated concentrations. 
